# Culvert Retrofit with Green Filter Media for the Removal of Phosphorus from Stormwater Runoff

**DOI:** 10.3390/ma19061193

**Published:** 2026-03-18

**Authors:** Somdipta Bagchi, Zhiming Zhang, Olayinka Olayiwola, Bharadwaj Mandala, Rupali Datta, Subhasis Giri, Richard Lathrop, Dibyendu Sarkar

**Affiliations:** 1 Department of Civil, Environmental and Ocean Engineering, Stevens Institute of Technology, Hoboken, NJ 07030, USA; somdiptaworkbagchi@gmail.com (S.B.); layiwolaolayinka@gmail.com (O.O.); 2 Office of Sustainability, Institute of Engineering & Management, Kolkata 700091, West Bengal, India; 3 Department of Civil and Environmental Engineering, Rowan University, Glassboro, NJ 08028, USA; zhangz@rowan.edu (Z.Z.); mandal63@rowan.edu (B.M.); 4 Department of Biological Sciences, Michigan Technological University, Houghton, MI 49931, USA; rupdatta@mtu.edu; 5 Center for Remote Sensing & Spatial Analysis, Rutgers University, New Brunswick, NJ 08901, USA; girisubh@msu.edu (S.G.); lathrop@crssa.rutgers.edu (R.L.)

**Keywords:** phosphorus contamination, stormwater runoff, adsorption, drinking water-treatment residuals, green engineering retrofit

## Abstract

Phosphorus is a ubiquitous contaminant in urban and agricultural landscapes. A retention basin located in the southern part of Barnegat Bay, New Jersey, was identified as receiving stormwater runoff with elevated phosphorus concentrations. The basin is surrounded by expanding urban development, contributing to the progressive degradation of water quality in the bay, which is already highly eutrophic. This study evaluated the effectiveness of a culvert retrofit with a green filter media composed of granulated-aluminum-based drinking water-treatment residuals (Al-WTR) and granular carbon (5:1 ratio, *w*/*w*) for the removal of phosphorus and suspended sediments from stormwater runoff. The performance of the filter media was assessed through water quality monitoring following runoff events over a 12-month period. The results indicated that the green filter media achieved up to 52% removal of total phosphorus from stormwater influent. However, treatment efficiency declined after approximately five months due to clogging of the geotextile bag housing the media. The replacement of the geotextile bag restored phosphorus removal performance (59%), highlighting the importance of routine maintenance. The findings demonstrate a cost-effective, environmentally sustainable, and innovative green engineering approach for mitigating phosphorus contamination in urban stormwater.

## 1. Introduction

Excess nutrients generated from anthropogenic activities such as agriculture, industry, and expanding urbanization contribute to the contamination of surface runoff, transporting nitrogen and phosphorus into water bodies. This nutrient enrichment promotes the growth of harmful algal blooms (HABs), resulting in oxygen depletion, ecosystem imbalance, and loss of biodiversity [1,2,3,4]. Although estuaries naturally receive metals and nutrients through geological weathering, a substantial portion of these loadings is anthropogenic, arising from fertilizers, soil erosion, wastewater discharges, vehicle emissions, and the release of organic materials such as animal waste, grass clippings, and leaf litter [5,6,7,8,9]. Urbanization further increases stormwater runoff volumes due to the expansion of impervious surfaces (e.g., parking lots, roads, and rooftops), which prevent water from infiltrating into the ground. This allows the excess phosphorus from lawns, gardens, and other urban sources to flow directly into surface water, seepage water, and eventually groundwater [10]. Changes in land use associated with deforestation, urbanization, and land conversion also degrade water quality by altering natural hydrology and reducing the capacity of ecosystems to retain and remove nitrogen and phosphorus [11]. Wastewater treatment plants and septic systems represent additional significant sources of nutrient inputs to water bodies. Moreover, atmospheric deposition of nitrogen and phosphorus from fossil fuel combustion, livestock operations, industrial activities, and conversion of perennial vegetation to row crop production further contributes to nutrient loading in aquatic systems [12,13,14]. Elevated nutrient concentrations, often accompanied by heavy metals and coliform bacteria from anthropogenic sources, accelerate eutrophication and subsequent deterioration of water quality [5,7,15]. Nutrient pollution of water in the U. S. imposes substantial economic and social costs, including reduced property values, increased water-treatment expenses, diminished recreational and aesthetic value, and financial losses in tourism and other water-dependent industries [16]. For instance, eutrophication in U.S. freshwater systems is estimated to cost approximately USD 2.4 billion annually, with recreational water use losses alone accounting for nearly USD 1 billion [17].

Barnegat Bay, a brackish coastal water body, is a small extension of the Atlantic Ocean in the U.S. Northeast. It is a nationally significant coastal system designated as the 28th site in the National Estuary Program by the U.S. Environmental Protection Agency (EPA) [18]. The Barnegat Bay watershed spans approximately 1709 km^2^ and includes more than 33 municipalities in Ocean County and 4 municipalities in Monmouth County, New Jersey [19]. Numerous tributaries, including the Toms River, Forked River, Metedeconk River, Cedar Creek, Oyster Creek, Mill Creek, Westecunk Creek, and Tuckerton Creek, drain into the bay, transporting nutrients and other contaminants that ultimately discharge into the Atlantic Ocean. The estuary is recognized for its rich marine life and ecological diversity and has long served as an important economic and environmental resource for the state. However, a baseline pollution assessment conducted at 15 locations within the Barnegat Bay–Little Egg Harbor system identified contaminants such as total petroleum hydrocarbons, mercury, polyaromatic hydrocarbons (PAHs), and polychlorinated biphenyls (PCBs) at concentrations posing potential risks to human health. In addition, nutrients including nitrogen and phosphorus were detected at levels exceeding recommended thresholds, contributing to fish kills, algal blooms, reduced dissolved oxygen concentrations, declines in clam populations, and increases in sea nettle jellyfish (*Chrysaora quinquecirrha*) populations in the bay [20,21]. Previous studies have shown that population growth and expanding urban and suburban development have significantly increased sediment and nutrient loading, particularly in the northern region of Barnegat Bay, while elevated nutrient inputs have also been observed in the central and southern portions of the bay [6,22,23].

Phosphorus removal from stormwater is a critical challenge, particularly for the protection of sensitive receiving waters such as Barnegat Bay. This necessitates the development of high-performance adsorptive filter media that are cost-effective and ideally, environmentally sustainable, and those that can be retrofitted into existing drainage infrastructures (e.g., culverts) without extensive capital investment or structural modification. High-capacity commercial media such as granular ferric hydroxide (GFH) and activated alumina demonstrate superior phosphate adsorption under typical stormwater conditions [24,25]. However, despite their high treatment efficiency under elevated loading conditions, their widespread implementation is constrained by high material costs, limiting scalability. These limitations have driven extensive research into low-cost alternatives, including drinking water treatment residuals, steel slag, and fly ash amendments, which have demonstrated 60–90% phosphorus removal while simultaneously promoting the beneficial reuse of industrial byproducts [26,27,28]. More recently, hybrid materials such as lanthanum-modified biochar have shown enhanced adsorption performance and pH resilience, although their long-term hydraulic performance under real-world stormwater conditions remains to be fully evaluated [29].

The primary objective of this study was to evaluate the field application of a green filter media primarily composed of non-hazardous, granulated-aluminum-based drinking water-treatment residuals (Al-WTR), produced through a patented process (US20200316556A1), for phosphorus removal from stormwater runoff. To achieve this objective, a retention basin within the Barnegat Bay watershed was selected based on prior water quality analyses indicating elevated phosphorus concentrations. A green filter medium consisting of granulated Al-WTR (a repurposed, non-hazardous industrial solid waste) and granular carbon was developed as a cost-effective and environmentally sustainable treatment solution. A retrofit device was designed and installed in an existing culvert directing stormwater from the retention basin to downstream surface waters, with the goal of reducing overall phosphorus loading.

## 2. Materials and Methods

### 2.1. Study Area

**Figure 1:**
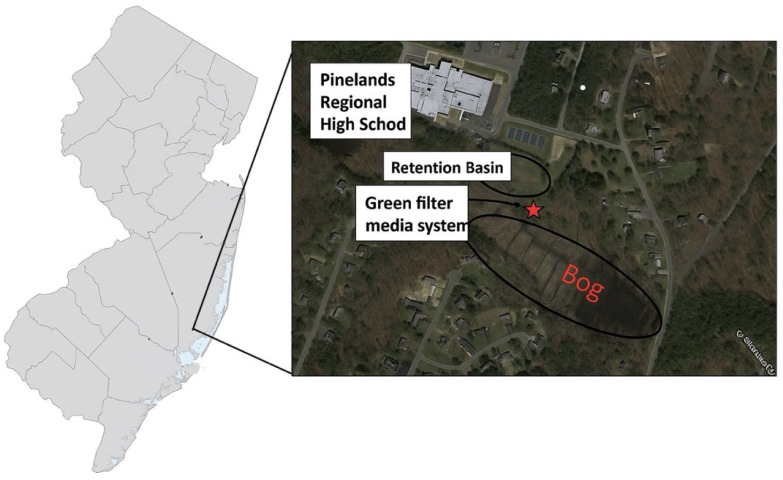
Aerial view of the study site and the location of green filter media.

This study was conducted in the southern portion of Barnegat Bay, New Jersey, USA (Figure 1). The retrofitted culvert is located adjacent to a retention basin at Pinelands Regional High School (Figure 1). Multiple rounds of stormwater sampling were conducted in the basin, and the results indicated elevated total phosphorus (TP) concentrations. Based on these findings, the site was selected as a demonstration location for water quality improvement using the innovative green filter media.

### 2.2. Chemicals

All chemicals used in this study were of reagent grade and were purchased from Sigma-Aldrich (St. Louis, MO, USA) and Fisher Scientific (Waltham, MA, USA). Deionized water was used for all solution preparation and analytical procedures throughout the study.

### 2.3. Preparation of the Green Filter Media

Al-WTR is a byproduct of the drinking-water-treatment process in which alum is used as the primary coagulant, which is typically treated as non-hazardous solid waste. A toxicity characteristic leaching procedure (TCLP) was conducted using the USEPA method 1311 [30] to ensure the non-hazardous property of the Al-WTR obtained from a local drinking-water-treatment facility in this study (Table S1). The effectiveness of Al-WTR in removing a range of pollutants, including common stormwater contaminants and heavy metals, has been previously reported [31]. The granular Al-WTR used in this study was produced through a patented repurposing process (US20200316556A1), which minimizes the use of hazardous reagents and reduces additional waste generation (Figure S1). The green filter media was prepared from granular Al-WTR and granular carbon. The granular carbon is approximately 2–3 mm in particle size, which was obtained in bulk from Hongtai Water Treatment Material Factory (Jiaozuo, Henan, China) [31].

Several ratios of granular Al-WTR to granular carbon (1:0, 5:1, 1:1, and 0:1) were evaluated, and an optimal composition for phosphorus removal was identified through laboratory batch experiments investigating adsorption kinetics. The hydraulic conductivity of the green filter media at each ratio was also determined following the protocol described by Na Nagara et al. [31].

### 2.4. Experimental Design and Setup

**Figure 2:**
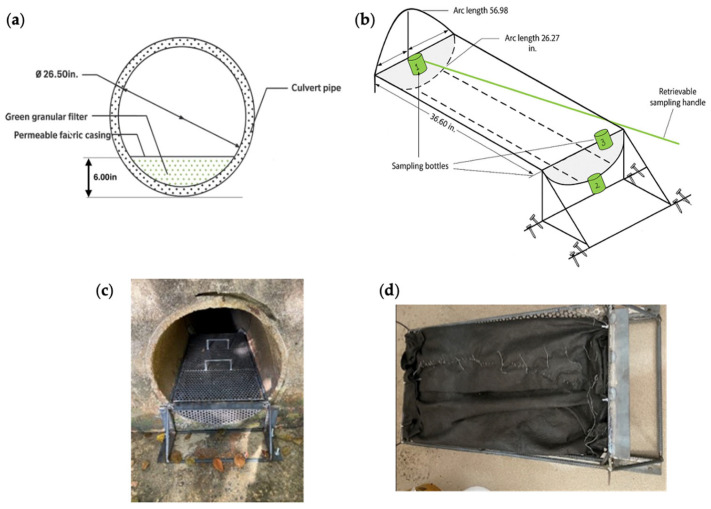
Schematic diagrams of (**a**) the culvert and (**b**) the framework installed in the culvert. Photographs of (**c**) framework installation and (**d**) green filter media sealed in a permeable fabric casing made of nonwoven geotextile.

The study site (Figure 1) is located at a retention basin adjacent to Pinelands Regional High School (39°36′ N, 74°21′ W) in New Jersey. Stormwater from the high school and surrounding areas drains into the retention basin and is conveyed through a culvert to Gifford Mills Branch Bog, which ultimately discharges into Barnegat Bay. The concrete pipe culvert is 19.8 m long with an internal diameter of 0.673 m and a headwall width of approximately 1.52 m (Figure S2). The green filter media was placed within a permeable fabric casing made of nonwoven geotextile and installed at the bottom of the culvert using a stainless-steel support framework acting as a retrofit device (Figure 2). The filter media depth was 0.152 m, providing effective filtration during typical storm events while minimizing flow obstruction during high-intensity rainfall events. The framework serves as a critical structural component, providing the necessary strength and stability to secure the geotextile casing and filter media in place. A retrievable sampling device was used to collect influent water samples prior to contact with the filter media. A second sampling bottle was installed downstream to collect effluent stormwater after filtration, allowing for comparison of water quality before and after treatment. During heavy storm events, overflow water was captured in an additional sampling bottle and analyzed for pollutant concentrations. All collected samples were refrigerated immediately after collection and transported to the laboratory for analysis.

After five months of operation, the spent filter media were collected and evaluated for the leaching potential of hazardous metals using a synthetic landfill leachate solution. The eight Resource Conservation and Recovery Act (RCRA 8) metals were quantified in the leachate using inductively coupled plasma optical emission spectrometry (ICP-OES; Model 5100, Agilent Technologies, Santa Clara, CA, USA). The phosphorus adsorption capacity of the spent filter media was also assessed through adsorption isotherm experiments. For the isotherm study, 40 mL phosphorus solutions were prepared at varying initial concentrations (0.05, 0.1, 0.2, 0.6, 1, 1.5, 2, 10, 20, 50, 75, 100, and 125 mg/L). The spent filter media were added at a 1% (*w*/*v*) solid-to-solution ratio. Desorption experiments were subsequently performed using the spent filter media from the isotherm study to assess the potential release of adsorbed phosphorus. All experiments were conducted in triplicate, and results are reported as mean values with corresponding standard deviations.

### 2.5. Sample Analysis

The collected water samples were analyzed for pH, TP, total nitrogen, nitrite, nitrate, phosphate, and total suspended solids (TSS). pH was measured using a benchtop multiparameter meter (PC700, Oakton, IL, USA). Total phosphorus concentrations were determined by ICP-OES following USEPA Method 3050B hot block acid digestion (USEPA, 1996) [32]. Total nitrogen was measured using HACH test kits based on the persulfate digestion Test ‘N Tube method (Method 10071). Concentrations of anions (nitrite, nitrate, and phosphate) were measured using ion chromatography (Dionex™ Integrion™ HPIC™ System, Thermo Fisher Scientific, Carlsbad, CA, USA), while ammonium concentrations were determined using ion chromatography (Dionex Aquion Ion Chromatography System, Thermo Fisher Scientific, Carlsbad, CA, USA). Total suspended solids (TSS) were quantified following Standard Method 2540D [33].

## 3. Results and Discussion

### 3.1. Stormwater Characterization

Characterization results of water samples collected from the retention basin prior to the culvert retrofit are presented in Table 1. The pH values were within the permissible range established by the New Jersey Surface Water Quality Standards (4.5–8.5), indicating neutral to slightly acidic conditions suitable for most aquatic ecosystems. Concentrations of nitrogen species were also below regulatory thresholds, suggesting that nitrogen pollution was not a primary concern at the sampling site. In contrast, the TP concentration was measured at 0.09 ± 0.04 mg/L, exceeding the applicable standard of 0.05 mg/L. This elevated phosphorus level indicates potential nutrient loading from surrounding areas, including the school premises, into the retention basin. Such nutrient enrichment increases the risk of eutrophication in downstream ecosystems, including Gifford Mills Branch Bog and ultimately Barnegat Bay.

### 3.2. Optimizing the Composition of Green Filter Media

**Figure 3:**
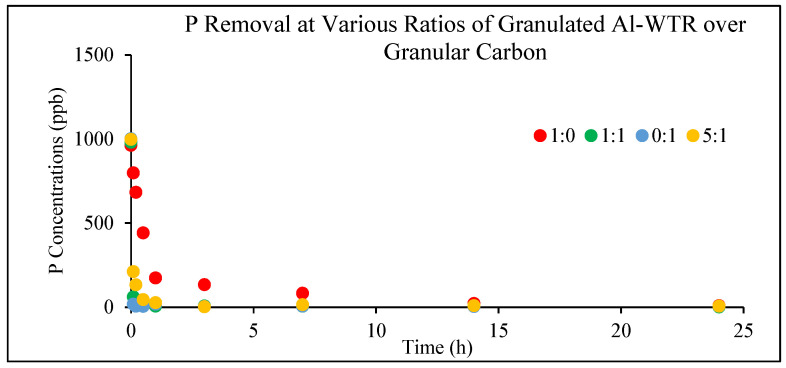
Phosphorus adsorption kinetics by filter media with different ratios of granulated Al-WTR over granular carbon (with 1% *w*/*v* solid to solution ratio, pH adjusted to 7 using HNO_3_ and NaOH, and room temperature at 23 ± 1 °C).

To optimize the composition of the green filter media for phosphorus removal, four different ratios of granular Al-WTR to granular carbon were evaluated: 1:0, 5:1, 1:1, and 0:1. The adsorption study was conducted at room temperature (23 ± 1 °C) with 1% *w*/*v* solid to solution ratio and pH adjusted to 7 using HNO_3_ and NaOH. The kinetics study showed that phosphorus was effectively adsorbed by all tested media compositions within the equilibration period (Figure 3). Notably, the addition of granular carbon significantly reduced the time required to reach adsorption equilibrium. When granular Al-WTR was used alone (1:0), more than 80% of phosphorus was removed within one hour. In contrast, at a 5:1 Al-WTR-to-carbon ratio, 80% removal was achieved within 6 min. An even higher removal efficiency of 94% within 6 min was observed for the 1:1 ratio, demonstrating the role of granular carbon in accelerating the adsorption process. Despite the improved removal kinetics, granular carbon exhibited substantially lower hydraulic conductivity (6.24 × 10^−6^ m/s) compared to granular Al-WTR (1.37 × 10^−3^ m/s), as shown in Table 2. Therefore, hydraulic conductivity was considered a critical parameter in optimizing the media composition for field-scale application. By balancing phosphorus removal efficiency with hydraulic performance, the 5:1 Al-WTR-to-carbon ratio was selected as the optimal green filter media composition.

### 3.3. Green Media Filtration System—Field Application

**Figure 4:**
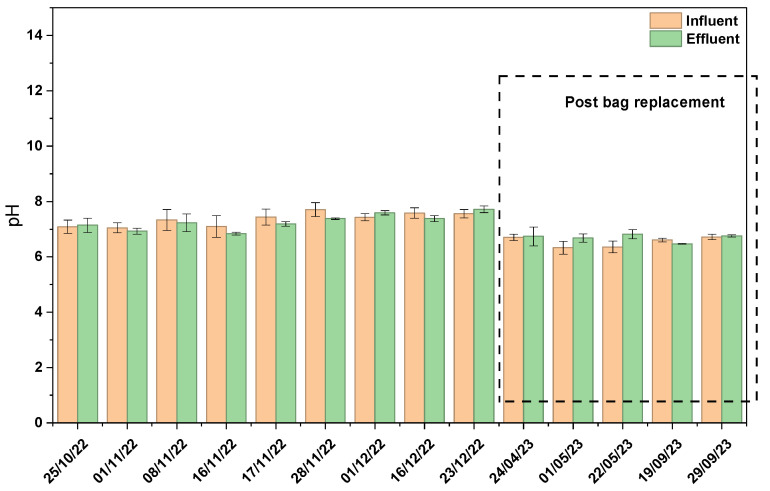
pH of influent and effluent stormwater samples from the green media filtration system.

The field study was conducted over one year, from October 2022 to September 2023, during which 22 sampling events were performed. Sampling was conducted at regular intervals, particularly following rainfall events. Both influent samples (prior to treatment) and effluent samples (after passing through the green filter media) exhibited pH values ranging from 6.36 ± 0.21 to 7.71 ± 0.25, while effluent pH ranged from 6.47 ± 0.02 to 7.59 ± 0.08 (Figure 4). The application of the filter media did not result in significant changes in pH. As reported in [34,35], pH plays a very important role in phosphorus adsorption on Al-WTR, as the mechanism is based on ligand exchange, where phosphate ions replace hydroxyl (OH) groups on amorphous AI–hydroxide surfaces. The optimum pH typically ranges between 5 and 7, i.e., mildly acidic–neutral, as the protonated surface due to its positive charge attracts negatively charged phosphate species. As pH increases, hydroxy ions increasingly compete with phosphate for adsorption sites, resulting in reduced electrostatic attraction due to surface deprotonation [34,35].

**Figure 5:**
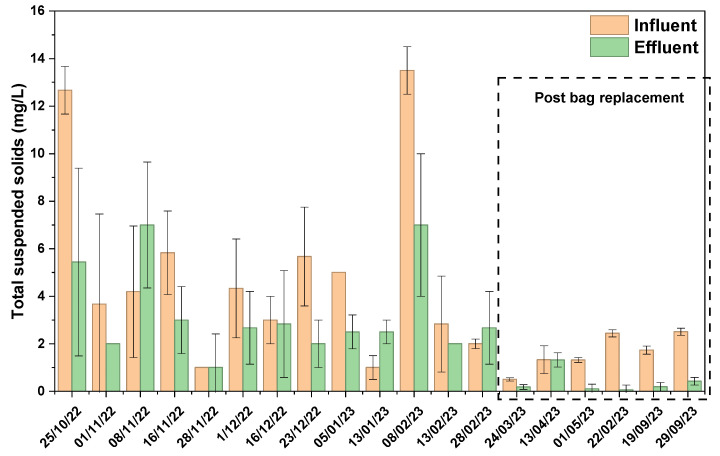
Total suspended solids of influent and effluent stormwater samples from the green media filtration system.

The TSS data of field analysis showed volatility, as on occasions negative removal was noticed, indicative of a phenomenon called “scouring”, where the accumulation of solids on the filter bed increases the pressure on the pores of the media dislodging previously trapped particles (Figure 5). The concentration varied from non-detectable levels to a high of 13.5 mg/L. In the early phase of operation, the system provided moderate solids removal (57.02 ± 32.8%). However, as the filter bag began to clog, the performance degraded, eventually leading to a catastrophic release. Negative removal efficiencies were recorded on days like 28th February (−33.33 ± 16.36%) attributed to flushing of stored material from the clogged filter. Following the bag replacement, the TSS removal efficiency showed immediate stabilization with a maximum reaching 97 ± 8.20%, representing a complete restoration of the filtration function of the media. A Mann–Whitney U test showed significant improvement in TSS removal efficiency (*p* < 0.0001) after bag replacement, with all post-replacement values ranked higher than pre-clogging.

**Figure 6:**
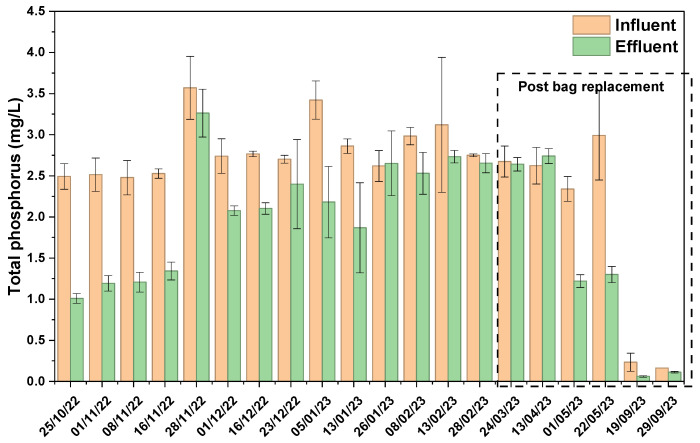
Total phosphorus of influent and effluent stormwater samples from the green media filtration system.

The assessment for influent phosphorus concentration remained relatively stable with an average of 2.84 ± 0.33 mg/L and ranging from 2.47 ± 0.2 mg/L to 3.57 ± 0.38 mg/L (Figure 6). The removal efficiency exhibited a downward trend from the month of December, signaling the gradual clogging of the bag. During the months of October and early November, removal efficiency ranging from 45.4 ± 0.15% to 52.53 ± 0.35% was demonstrated, following which a sharp decline in performance efficiency was observed. By March and April, the system’s ability to remove phosphorus had nearly vanished, and lowest percentage removal of −4.44 ± 1.18% was recorded. After replacement of the bag, the phosphorus removal efficiency sharply improved, and the highest reported efficiency was 59 ± 1.2%. Bag replacement was an effective strategy for maintaining filtration efficiency. The Mann–Whitney U test (*p* = 0.15) confirmed an insignificant statistical difference in TP removal efficiency between pre and post replacement, confirming the highest achievable removal efficiency in this setup as 59%. Previous studies have demonstrated effective phosphorus removal using Al-WTR, with amorphous aluminum oxides and hydroxides playing a critical role in sorption processes [36]. Babatunde et al. [37] reported a maximum phosphorus adsorption capacity of 31.9 mg/g for Al-WTR, while Rahmati et al. observed a comparable value of 28.7 mg/g [36,37]. Concentrations of total nitrogen, ammonium, nitrate, and nitrite were also monitored in influent and effluent samples. All measured values remained below the New Jersey Surface Water Quality Standards limits, indicating that nitrogen species were not a primary contaminant of concern at the study site.

The TCLP test was conducted to evaluate the leaching potential of hazardous metals and phosphorus from the spent green filter media after five months of operation. Some RCRA-regulated metals, including As, Cd, Cr, and Hg, were detected in the leachates; however, their concentrations were well below USEPA regulatory limits (Table 3). These findings suggest strong retention of both nutrients and heavy metals by the filter media, indicating that the spent media may be disposed of in a conventional landfill without requiring additional treatment.

### 3.4. Phosphorus Adsorption Analysis on the Spent Filter Media

To evaluate the continued functionality of the filter media, phosphorus adsorption analyses were performed on the spent media after five months of operation. The adsorption behavior of phosphorus on the spent green filter media was assessed using adsorption isotherms. Langmuir, Freundlich, and Redlich–Peterson isotherm models were applied to fit the experimental data (Figure S3). The results indicate that the Redlich–Peterson isotherm provided the most accurate representation of phosphorus behavior on spent filter media, indicating a complex adsorption mechanism with heterogeneous surface characteristics [36]. Furthermore, phosphorus desorption experiments confirmed the irreversible adsorption of phosphorus onto the spent filter media. Across initial concentrations ranging from 0.05 mg/L to 50 mg/L, percentage desorption remained negligible (0–0.16%), with only trace release from the highest concentration of 50 mg/L, indicating strong bonding rendering the media suitable for long-term P sequestration.

### 3.5. Comparative Analysis with Other Field-Scale Studies

Several field- and column-based studies have reported phosphorus removal using Al-WTR and WTR amended media from urban runoff. For example, a column study with media amended with sandal-WTR reported consistently more than 90% phosphorus removal and significantly higher than the unamended control. In an engineered stormwater filtration system using granular drinking-water-treatment residuals, phosphate removal efficiency of more than 86% was achieved in controlled column studies, highlighting the potential of drinking-water-treatment residuals in effective P capture. Field experiments with bioretention cells retrofitted with water-treatment residuals also reported a significant reduction in TP, even under large storm event conditions. The comparatively lower treatment efficiency achieved in the current study can be attributed to the differences in hydraulic conditions, influent variability, and maintenance regimes under real field conditions, further highlighting the importance of continuous optimization of media design and operational parameters to improve the performance of the filtration setup [38,39,40].

### 3.6. Sustainability Implications

The use of green filter media for stormwater treatment offers significant advantages from a sustainability perspective. This approach supports sustainable waste management and aligns with circular economy principles by repurposing waste-derived materials such as aluminum-based water-treatment residuals (Al-WTR), which would otherwise be disposed of in landfills [37,40,41]. By converting industrial solid waste into a functional water-treatment material, this process promotes resource recovery and waste valorization. Poor et al. demonstrated the successful use of Al-WTR in green roof retrofits to reduce phosphorus leaching, highlighting its effectiveness when properly maintained [42]. These findings support the broader applicability of Al-WTR in urban stormwater management systems within circular economy frameworks. Al-WTR and similar materials are abundant, low-cost, and locally available, making them practical and accessible options for improving urban water quality [34,42,43,44]. Compared with conventional chemical-based adsorbents, which are often expensive and environmentally burdensome, green filter media represent a more environmentally friendly alternative with a reduced ecological footprint. Their application decreases reliance on synthetic treatment chemicals and lowers carbon emissions associated with material production and disposal, making them a compelling option for sustainable urban stormwater management.

## 4. Conclusions

A retention basin within the Barnegat Bay watershed of New Jersey was identified to be exhibiting elevated phosphorus concentrations in stormwater, a key driver of eutrophication in receiving waters. Because the basin discharges into Gifford Mills Branch Bog, which ultimately drains into Barnegat Bay, reducing phosphorus loading at this upstream location is important for mitigating eutrophication in the bay. This study evaluated a retrofitted green filtration system installed within the culvert connecting the retention basin to the bog, designed to capture phosphorus from stormwater runoff. A green filter media, composed of granulated-aluminum-based water-treatment residuals (Al-WTR) and granular carbon at a 5:1 ratio, achieved up to 59% removal of total phosphorus over a 12-month monitoring period. However, treatment efficiency declined over time due to clogging of the geotextile bags housing the filter media at the base of the culvert. Following bag replacement, the phosphorus removal efficiency substantially improved, reaching 59%. Total suspended solids removal efficiency also increased significantly after bag replacement, with efficiencies approaching 97%. No notable changes were observed between influent and effluent pH. The TCLP results confirmed that metal leaching from spent filter media remained well below regulatory limits, supporting safe disposal. These observations indicate that green filter media made from water-treatment residuals can provide an effective, low-cost, and sustainable strategy for reducing phosphorus loading in urban stormwater systems, although routine inspection and maintenance of the filtration unit are mandatory for sustained performance.

## Figures and Tables

**Table 1:** Characterization of water from the retention basin.

Parameters	Retention Basin Water Samples	New Jersey Surface Water Quality Standards
Ammonium-N (mg N/L)	BDL ^1^	~1.30
Nitrate-N (mg N/L)	0.99 ± 0.12	2.00
Total Nitrogen (mg N/L)	1.80 ± 0.30	N/A
Total Phosphorus (mg P/L)	0.09 ± 0.04	0.05
Total Suspended Solids (mg/L)	0.36 ± 0.13	25.00
Average pH	7.18 ± 0.14	4.50–8.50

Note: ^1^ BDL = below detection limit.

**Table 2:** Hydraulic conductivity performance of the filter media with different ratios of granulated Al-WTR over granulated carbon.

Granulated Al-WTR: Granular Carbon	Hydraulic Conductivity (m/s)
1 to 0	1.37 × 10^−3^
1 to 1	1.20 × 10^−5^
5 to 1	4.03 × 10^−5^
0 to 1	6.24 × 10^−6^

**Table 3:** Toxicity characteristic leaching procedure (TCLP) values (µg/L) of spent green filter media.

TCLP	As	Ba	Cd	Cr	Pb	Hg	Se	Ag	P
**Spent Green Filter Media**	8.22 ± 1.78	BDL ^1^	0.98 ± 0.07	12.92 ± 0.15	BDL	34.24 ± 20.33	BDL	BDL	0.09 ± 0.01
**USEPA Limit**	5000	100,000	1000	5000	5000	200	2000	5000	50

Note: ^1^ BDL = below detection limit.

## Data Availability

The original contributions presented in this study are included in the article/supplementary material. Further inquiries can be directed to the corresponding author.

## Supplementary Materials

The following supporting information can be downloaded at: https://www.mdpi.com/article/10.3390/ma19061193/s1, Figure S1: Transformation of raw Al-WTR to granulated form; Figure S2: General overview of culvert structure and dimensions where water flows from the retention basin to the Gifford Mills Branch Bog; Figure S3: Langmuir and Freundlich isotherms models representing phosphorous adsorption by spent filter media; Table S1: Toxicity characteristic leaching procedure (TCLP) values (µg/L) of Al-WTR.

## Abbreviations

The following abbreviations are used in this manuscript:

Al-WTR	aluminum water-treatment residual
NJ	New Jersey
TCLP	toxicity characteristic leaching procedure
USEPA	United States Environmental Protection Agency
RCRA	Resource Conservation and Recovery Act
TP	total phosphorus
TSS	total suspended solids
BDL	below detection limit
ICP-OES	inductively coupled plasma optical emission spectrometry
EPA	Environmental Protection Agency
NJDEP	New Jersey Department of Environmental Protection
PCB	polychlorinated hydrocarbons
PAH	polyaromatic hydrocarbons
HAB	harmful algal blooms
