# Supplementary material for: Culvert Retrofit with Green Filter Media for the Removal of Phosphorus from Stormwater Runoff
Source: Materials (Basel). 2026 Mar 18;19(6):1193. doi: 10.3390/ma19061193 (PMC13028067; doi:10.3390/ma19061193)
Supplement: Supplementary file 1 [file materials-19-01193-s001.zip › materials-4169644-supplementary.pdf]

Supplementary Materials

Culvert Retrofit with Green Filter Media for the Removal of Phosphorus from Stormwater Runoff

Somdipta Bagchi [1,2], Zhiming Zhang [3], Olayinka Olayiwola [1], Bharadwaj Mandala [3], Rupali Datta [4], Subhasis Giri [5], Richard Lathrop [5] and Dibyendu Sarkar [1,*]

[1] Department of Civil, Environmental and Ocean Engineering, Stevens Institute of Technology, Hoboken, NJ 07030, USA
[2] Office of Sustainability, Institute of Engineering & Management, Kolkata 700091, West Bengal, India
[3] Department of Civil and Environmental Engineering, Rowan University, Glassboro, NJ 08028, USA
[4] Department of Biological Sciences, Michigan Technological University, Houghton, MI 49931, USA
[5] Center for Remote Sensing & Spatial Analysis, Rutgers University, New Brunswick, NJ 08901, USA
* Correspondence: dsarkar@stevens.edu

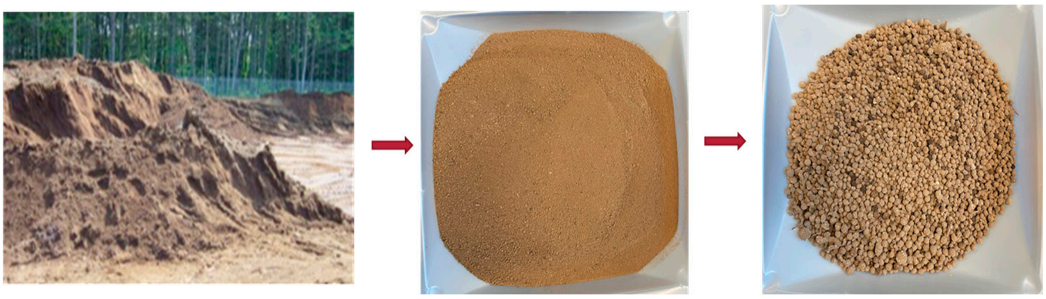

Figure S1. Transformation of raw Al-WTR to granulated form.

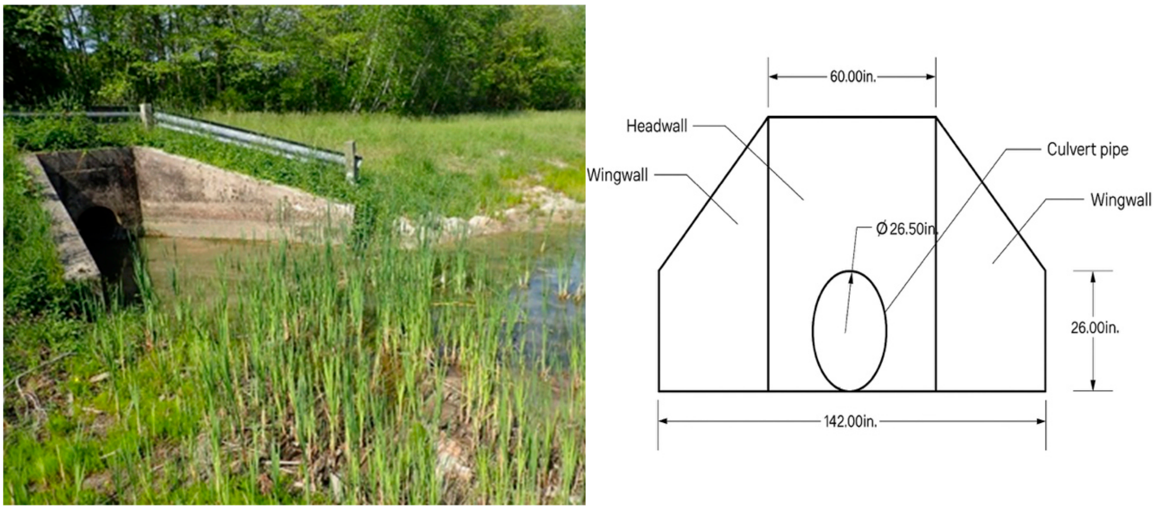

Figure S2. General overview of culvert structure and dimensions where water flows from the retention basin to the Gifford Mills Branch Bog.

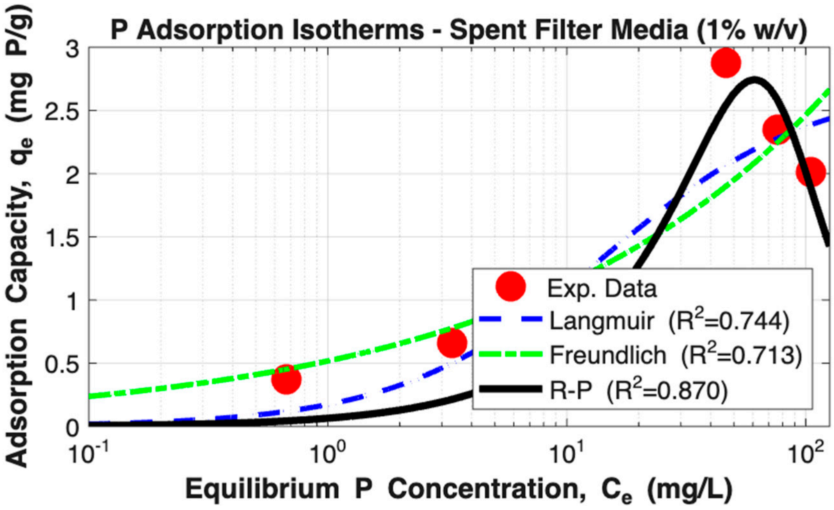

Figure S3. Langmuir and Freundlich isotherms models representing phosphorous adsorption by spent filter media.

Phosphorus adsorption isotherm study on spent filter media was conducted using Langmuir, Freundlich, and Redlich-Peterson models across different concentrations. The Redlich-Peterson model provided the superior fit with R^2 = 0.870 and lowest root mean square error (RMSE) = 0.3322 with equation shown below:

$$q_e = \frac{K_{RP} C_e}{1 + a_{RP} C_e^{\beta}} \tag{1}$$

where, K_{RP} (L/g): Redlich-Peterson isotherm constant = 0.0646 L/g, a_{RP} (L/mg): Redlich-Peterson isotherm constant = 5.93x10^{-7}, β: exponent parameter = 3.28.

The Langmuir model provided moderate fit (R^2 = 0.7444, RMSE = 0.4653) with equation as:

$$q_e = \frac{q_m K_L C_e}{1 + K_L C_e} \tag{2}$$

where, q_m (mg/g): Maximum monolayer adsorption capacity = 2.72 mg/g, K_L (L/mg): Langmuir equilibrium constant related to binding energy = 0.068 L/mg.

The Freundlich model provided the weakest fit (R^2 = 0.7127, RMSE = 0.4933) with equation as shown below:

$$q_e = K_F C_e^{1/n} \tag{3}$$

where, K_F: Freundlich constant indicating adsorption capacity = 0.52, n: Heterogeneity factor (dimensionless) $n = 2.95$ indicating favourable adsorption.

While, the model fit of the Langmuir model suggests mechanism of monolayer adsorption on specific active sites, likely associated with surface complexation reactions, the Freundlich model (R^2 = 0.7127) indicates presence of surface heterogeneity and variable adsorption energies. The superior performance of the Redlich–Peterson model indicates that the adsorption process cannot be described solely by monolayer adsorption but involves a combination of heterogeneous surface interactions and concentration-dependent adsorption behaviour. Overall, this suggests that phosphorus removal is occurring through a combination of surface complexation, electrostatic adsorption, and possible precipitation reactions on heterogeneous adsorption sites.

Table S1. Toxicity characteristic leaching procedure (TCLP) values (μg/L) of Al-WTR.

RCRA Metals	Al-WTR	USEPA Limit
**As**	42.27±8.06	5000
**Ba**	976.15±39.29	100000
**Cd**	BDL [1]	1000
**Cr**	8.05±0.83	5000
**Pb**	13.81±1.02	5000
**Hg**	10.50±4.05	200
**Se**	BDL	2000
**Ag**	BDL	5000

Note: [1] BDL=Below Detection Limit.
